# Conference highlights of the 5^th ^international workshop on HIV persistence during therapy, 6-9 December 2011, St. Maartin, West Indies

**DOI:** 10.1186/1742-6405-9-7

**Published:** 2012-03-12

**Authors:** Mario Stevenson, Nicolas Chomont, Alain Lafeuillade

**Affiliations:** 1 Division of Infectious Diseases, University of Miami Leonard M. Miller School of Medicine, Two Story Lab (TSL), 1420 NW 9th Avenue (T-190), Room 109, Miami, Fl 33136, USA; 2 Vaccine and Gene Therapy Institute, 11350 SW Village Parkway, Port St Lucie, Fl 34987, USA; 3 Department of Infectious Diseases, General Hospital, 1208 avenue Colonel Picot, 83056, Toulon, France

**Keywords:** HIV persistence, HIV reservoirs, HIV latency, HIV cure, HIV eradication, HIV reservoir group

## Abstract

The December 2011 5^th ^International Workshop on HIV Persistence during Therapy addressed the issue of HIV persistence among 210 scientists from 10 countries involved in the study of HIV reservoirs and the search of an HIV cure. High quality abstracts were selected and discussed as oral or poster presentations. The aim of this review is to distribute the scientific highlights of this workshop outside the group as analyzed and represented by experts in retrovirology, immunology and clinical research.

## Introduction

The 5^th ^international workshop on HIV persistence during therapy was held in St. Maarten from December 6-9 and featured presentations from 210 scientists representing approximately 10 countries. Since its inception, the goal of the workshop has been to provide a forum for research aimed at understanding the mechanism by which HIV-1 persists in the face of antiretroviral therapy (ART) and to develop strategies with which to curtail viral persistence and accelerate the objective of viral eradication. While ART has fundamentally impacted the health of individuals living with HIV infection and effects durable suppression of plasma viral RNA to undetectable levels, current treatment regimens are unable to eradicate the virus [1]. In addition, pathogenic manifestations of HIV-1 infection are manifest despite potent viral suppression. Therefore, it is clear that we have to look beyond long-term maintenance of HIV-1 infection and ultimately develop strategies for viral eradication.

## Virology of HIV persistence

### Models of HIV latency

The development of strategies to eliminate HIV-1 reservoirs that persist in the face of ART will require a complete understanding of the nature of viral persistence and latency and how these processes are regulated. While latency is considered the single biggest obstacle to viral eradication, how latency is established and regulated is still not well understood. Most of the studies conducted to date on viral latency have focused on models that employ established cell lines. They have demonstrated various forms of latency regulation and the role of host cell cycle, epigenetic effects and other host cell factors that can regulate viral latency. However, it is unclear as to the extent to which these cell line models of viral latency reflect the true nature of latency as it exists in memory CD4^+ ^T cells [2]. For this reason, several investigators have attempted to develop primary cell models of viral latency so as to gain greater physiologic insight into viral persistence and latency and more importantly, how latency can be interrupted. *Planelles et al. *[3] described a system for the establishment of latency in *in vitro *differentiated central memory cells. The system uses peripheral blood mononuclear cells from healthy human donors. Following isolation of naïve CD4^+ ^cells by negative selection, cells are activated with CD3 and CD28 antibodies in medium containing TGF-beta, anti interleukin (IL)-12 and anti IL4 antibodies followed by culture in IL-2. Following HIV-1 infection, cells return to a quiescent state where the majority of infected cells are latent. *Planelles et al*. have previously demonstrated that these latently infected cells can be re-activated by incubation with CD3/CD28 antibodies. The investigators went on to use this system to screen for anti-latency agents. The investigators demonstrated that incubation with IL-2 + IL-7 can induce homeostatic proliferation and reactivation in about 10% of the latently infected cells. However, full reactivation of all latently infected cells in the cultures required antigenic stimulation. While this study describes a system that can be very useful for the screening of anti-latency agents, it highlights the concern that agents inducing homeostatic proliferation may have the potential to expand integrated proviruses through gene duplication at mitosis. Nonetheless, this won't impact the majority of latent genomes that fail to become reactivated during homeostatic proliferation and therefore, not achieve the goal of eradicating latent provirus. An important point to consider is whether homeostatic proliferation favors the duplication of archival and defective provirus rather than latent provirus. It is unclear whether latent provirus duplication by mitosis during homeostatic proliferation leads to expression of the provirus and subsequent clearance by cytopathic effects or by immune clearance. Defective provirus would be less likely to be cleared by these mechanisms. Therefore it would be important to determine to what extent homeostatic proliferation allows duplication of latent provirus without subsequent clearance.

Continuing on with the theme of primary models of viral latency, *Garcia et al. *[4] described the generation of HIV latency in BLT humanized mice. Infected BLT mice were administered an anti-retroviral regime comprising daily injections of FTC, tenofovir and raltegravir. Resting cells obtained from BLT mice on this anti-retroviral regime could be stimulated *ex vivo *to produce virus. The frequency of latently infected, resting cells was determined to be in the reaching of 9.9 per million CD4^+ ^T cells, which is in the range observed for patients on suppressive therapy. The availability of an *in vivo *model of viral latency extends the tools available for the analysis of anti-latency drugs.

### Role of myeloid cells

While the majority of research has focused on the role of lymphoid reservoirs in viral persistence and latency, several presentations focused on the role of myeloid cells in viral persistence. A large body of research has demonstrated the important association of animal lentiviruses with myeloid-lineage cells and, in particular, macrophages. However, very little attention has focused on the role of myeloid-lineage cells in the biology of primate lentiviruses. Early studies demonstrated infection of macrophage in the tissues such as the lung and central nervous system (CNS) but, beyond that, there is no clear understanding of whether macrophage support viral persistence in patients on antiretroviral therapy or whether latency is also manifest in macrophage or other myeloid-lineage cells. Arguably, the strongest piece of experimental evidence supporting an essential role for macrophage in primate lentivirus biology is the demonstration that myeloid-lineage cells harbor an antiviral restriction that is not exhibited by T cells and further, that the virus has evolved a strategy to circumvent this restriction.

*Clements et al. *[5] discussed the role of macrophage in establishment of viral reservoirs in the brain and spleen in SIV infected macaques on a 4-drug antiretroviral regime that provides a model for the study of viral persistence in the CNS. While ART was able to suppress viral replication in the brain, viral DNA was found to persist. It remains to be determined as to the extent of macrophage involvement in the persistence of viral DNA and whether a resident population of microglial cells constitutes a viral reservoir or whether perivascular macrophages, infected in the periphery, migrate to the CNS to maintain the viral reservoir. This theme was continued by *Russell et al. *[6] who examined involvement of splenic macrophages as well changes in this population during SIV infection following antiretroviral treatment. Macaques on a 4-drug antiretroviral regime recreated the dynamics of suppression of plasma viremia in patients on ART and also recreated low-level residual viremia. The data was consistent with the contribution of infected macrophages to this residual viremia in animals undergoing antiretroviral suppression. This promises to be a very important model for the study of viral persistence during therapy since it allows access to tissue reservoirs that are not accessible in patients and, in particular, will allow an investigation of the role played by the CNS in viral persistence.

## Immunology of HIV persistence

### Establishment of HIV reservoirs

In addition to the viral mechanisms of HIV-1 persistence, it is well established that the immune system plays a critical role in the establishment and persistence of a viral reservoir during therapy [7]. Interestingly, both the innate and adaptive arms of the immune system may contribute to HIV persistence during ART. As innate immune mechanisms fail short in controlling HIV replication during acute infection, HIV disseminates and infects antigen-specific T cells and macrophages in lymphoid organs where it establishes its reservoir and persists for years. Understanding the shortcoming of the innate immune response is therefore important to delineate the cascade of events leading to HIV persistence. *Lewin et al. *[8] presented insight into how chemokines play a role in the establishment and maintenance of HIV-1 latency. The investigators demonstrated that resting CD4^+ ^T-cells incubated with CCL19 and CCL21 receptor chemokines, CXCL 10 and CCR 6 rendered them permissive to HIV-1 infection and provirus establishment. Incubation with CCL 19 prior to infection activated RhoA signaling and alteration of the actin network that was sufficient for establishment of the integrated provirus. Furthermore, integration of HIV-1 in resting cells was also dependent upon PI3K pathway activation and inhibitors of the PI3K pathway did not affect nuclear localization of viral cDNA but prevented cDNA integration. *Lewin et al*. went on further to describe how dendritic cells may play a role in the infection of resting memory CD4^+ ^cells. Monocyte-derived dendritic cells (myeloid DCs) promoted latent infection of resting memory CD4^+ ^cells when in co-culture but less efficiently when cells were incubated separately. This suggests the presentence of soluble factors that may play a role in the promotion of CD4 latency by myeloid DCs.

### Innate immunity

*Lieberman et al. *[9] outlined that although HIV introduces many foreign nucleic acids into the cytosol, HIV infection of T cells and macrophages does not trigger an interferon (IFN) response. She proposed to identify the mechanisms by which HIV escapes detection in these cell subsets. She identified Trex1 (Three Prime Repair Exonuclease I) as a potent inhibitor of IFN induction during HIV infection of T cells and macrophage. Trex-1 digests viral reverse transcripts and in its absence, HIV DNA stimulated Type I IFN production. By using CD4 aptamer-siRNA chimeras that selectively knockdown gene expression in CD4^+ ^T cells, monocytes and macrophages, she demonstrated that the knockdown of CCR5, HIV Vif and gag or Trex1 inhibit HIV transmission in tissue explants and humanized mice.

*Laguette et al. *[10] discussed how the anti-viral restriction, recently identified as SAMHD1, influences the interplay between primate lentiviruses and myeloid cells. The genomes of primate lentiviruses are distinguishable from their animal retrovirus counterparts by the presence of additional small open reading frames that encode accessory proteins including Vif, Vpu, Nef and Vpr/Vpx. An explosion of research in the past several years has revealed that these accessory proteins form an antiviral defense against cellular antiviral proteins commonly referred to "antiviral restriction factors". Vif antagonizes the antiviral action of the Apobec3 cytidine deaminases, Vpu antagonizes the antiviral action of tetherin/BST 2 and, in viruses lacking a Vpu gene (HIV-2 and most SIV variants) tetherin is antagonize by Nef. For several years, it was apparent that the Vpr/Vpx proteins of primate lentiviruses specially enhance viral infection in myleoid-lineage cells and studies focusing on the Vpx protein of HIV-2/SIV demonstrated that myeloid-lineage cells including monocytes, macrophage and dendritic cells harbor a restriction that is counteracted by Vpx. In the past year, research from 2 groups identified the restriction as a deoxynucleoside triphosphate triphosphohydreolase called SAMHD1 [11,12]. SAMHD1 exerts a very potent antiviral effect on primate lentiviruses such that infection of myeloid-lineage cells by SIV is absolutely dependent upon a functional Vpx gene. Intriguingly, although HIV-1 is antagonized by SAMHD1, the Vpr gene of HIV-1 does not appear to have the capacity to neutralize this restriction. Nevertheless, HIV-1 can establish myeloid cell infection in the face of SAMHD1 restriction. Therefore, it remains to be determined whether HIV-1 possesses some degree of resistance to SAMHD1 restriction and whether this property impacts its ability to persist within myeloid cell reservoirs. At least, in the case of HIV-2/SIV, establishment of a macrophage reservoir is dependent upon neutralization of SAMHD1 by Vpx. Since primate lentiviruses have evolved a strategy to circumvent SAMHD1, and since this restriction is not exhibited by lymphoid cells, it argues that primate lentiviruses must occupy myeloid-lineage cells for some reason and it is tempting to speculate that myeloid-lineage cells contribute to the persistence nature of primate lentivirus infection.

*Manel et al. *[13] gave further insights into the important role of DCs during HIV infection. DCs posses the machinery to sense HIV-1 but the restriction imposed by SAMHD1 prevents optimal sensing. Thus, restoring HIV-1 replication in DCs by using Vpx may trigger innate sensing, thereby promoting HIV-specific immunity and inhibiting the infection of T cells in trans through the production of type I IFN.

*O'Doherty et al. *[14] quantified HIV molecular forms in Elite Controllers (EC), a rare population of subjects who naturally control HIV replication. EC displayed low levels of integrated DNA and high levels of unintegrated DNA, suggesting a possible defect in HIV integration as recently reported [15,16]. She further investigated the ability of CTLs from these subjects to control the size of the HIV reservoir by using an *in vitro *assay. Her data suggested that latently infected cells continuously express low levels of viral proteins and constitute targets for HIV-specific T cell responses. In an *in vitro *killing assay, EC displayed more effective removal of latently infected CD4^+ ^T cells than chronic progressors.

*Lichterfeld et al. *[17] also presented data pertaining to the susceptibility of CD4^+ ^T cells from EC to HIV-specific CD8^+ ^T cell mediated killing. In a cytotoxicity assay, CD4^+ ^T cells from EC showed an increased susceptibility to CD8 mediated killing. Interestingly, a reduced susceptibility of naïve CD4^+ ^T cells to CD8^+ ^T cells mediated killing was associated with a lower viral reservoir in EC.

*Deeks et al. *[18] focused on the role of residual levels of immune activation in HIV persistence during ART [19]. Inflammatory biomarkers (hsCRP, IL-6 and D-Dimer) as well as activation markers on CD8 T cells remained elevated during ART when compared to uninfected controls. While T cell activation levels and cell-based measures of viral persistence (proviral DNA and viral RNA) were weakly associated in the blood, the association between these factors was much stronger in gut mucosa. *Deeks et al. *summarized the recent results from two Raltegravir intensification trials indicating that this intervention did not modify the levels of T cells activation in blood and rectum but may reduce them in the terminal ileum [20,21].

*Chomont et al. *[22] listed behavioral and clinical parameters associated with the HIV reservoir size (CD4 and CD8 counts, age, route of transmission). In a multivariate analysis, only CD4^+ ^T cell nadir significantly predicted levels of HIV proviral DNA. The evolution in the TCR repertoire of virally suppressed subjects was correlated with the genetic evolution of the viral reservoir, supporting a model in which the dynamic of the memory CD4^+ ^T cell compartment drives the dynamic of the latent reservoir. In the second part of his talk, *Chomont *presented a novel assay aimed at monitoring HIV persistence during ART. The assay, which uses authentic CD4^+ ^T cells from virally suppressed subjects, could also be used to identify novel compounds aimed at reactivating HIV production in latently infected cells.

*Vandergeeten et al. *[23] outlined that IL-7 and IL-15 are involved in the maintenance of the pool of memory CD4^+ ^T cells and hypothesized that they may also contribute to the persistence of latently infected cells. Both cytokines induced proliferation, activation and survival of CD4^+ ^T cells *in vitro*. Similarly, both IL-7 and IL-15 enhanced viral production in productively infected CD4^+ ^T cells isolated from HIV infected subjects. Strikingly, the two cytokines differed in their ability to induce HIV production in latently infected cells, with IL-15 being much more efficient than IL-7. These results suggested that IL-15 should be considered as a possible candidate to force viral expression of the latent reservoir to achieve HIV eradication.

*Chirullo et al. *[24] started their presentation by summarizing the main results of a recently published article [25] showing that the gold-based drug auranofin can reduce the size of the SIV reservoir by downregulating the CD27 receptor. Auranofin induced both phenotype changes and cell-death, which were more pronounced in the memory compartment. The effects of auranofin were enhanced by Buthionine Sulfoximine, a drug used in chemotherapy that reduces the levels of glutathione. This strategy could be used to decrease the lifespan of the latently infected cells thus restricting the viral reservoir size [26].

## Innovative therapeutic strategies

### Designing eradication trials

*Kuritzkes et al. *[27] mentioned that although a number of promising approaches have led to testable hypotheses for an HIV cure, for which small animal and primate models may provide preliminary information, ultimately proof of concept and validation studies in human subjects will be required. This leads to determine the nature of end-points to be included in eradication trials (quantitative measures of HIV reservoirs, in blood and tissues) and the requirement for analytic treatment interruption. This one is the most rigorous way to assess a functional cure (no viremia rebound) but is not without risks. Finally, it is also important to define in which kind of patients these trials will first be proposed: patients on suppressive ART (which line?), well preserved immune functions (EC?) or advanced disease, patients with malignancies? This may depend of the outcome and the potential risks of the strategies. However, it is challenging to define an acceptable level of risk in the context of generally safe and effective life-long therapy. If a functional cure could be achieved with a period of treatment with some toxic drugs, how much toxicity would be acceptable? This could go from a small fatal risk (as to pancreatitis with ddI) to a modest risk (as for coronary artery bypass grafting: 1-2%) or a significant risk (like with bone marrow transplants: 10-20%).

It will undeniably be concerted decisions to make between patients scientists, ethic committees and funding agencies.

### ART at acute HIV infection

The immunological and virological benefits of ART initiation during the early steps of HIV infection have been reported for more than 10 years [28,29]. Starting ART during the first weeks of infection is associated with a reduced magnitude of the HIV reservoir after prolonged treatment. Importantly, a recent study indicates that early and prolonged ART can even lead to a natural and prolonged control of the viremia after treatment interruption [30]. Preliminary results from two studies aimed at identifying precisely the benefits and the mechanisms by which early treatment limits the seeding of the reservoir were presented at this workshop.

*Markowitz et al. *[31] presented the 96 weeks results of their single-site study of 3- vs. 5-drug therapy in early/acute HIV infection. They compared Tenofovir/Emtricitabine plus Atazanavir/ritonavir or Darunavir/ritonavir to the same agents plus Raltegravir and Maraviroc.

At 48 weeks, from 26 patients randomized in the 5-drug arm, 23 patients remained on study; and from 14 patients randomized to the 3-drug arm, 11 patients remained on study.

At 96 weeks, 18 patients remained on study in the 5-drug arm, and 10 patients in the 3-drug arm. As expected the 5-drug regimen achieved < 50 copies/ml of plasma viremia faster, but at week 48 there were 3 virological failures in the 5 drug arm, and none in the 3-drug arm. No differences were found in terms of proviral DNA levels or infectious virus titers between the 2 arms. Overall, these results are quite disappointing but concern a population of patients treated after a mean duration of symptoms of acute infection of about 50 days.

On the contrary, *Ananworanich et al. *[32] treated truly cases of acute infection. Their protocol selection allowed to diagnose 74 patients, and to include 62 of them within a few days after infection. From the 35 patients who were analyzed at the time of the presentation, 12 were treated at Fiebig stage I (5 days after the eclipse phase), 3 at Fiebig II (10 days) and 17 at Fiebig III (13 days). The ART regimens were Tenofovir/Emtricitabine/Efavirenz in 13 patients and the same regimen + Raltegravir and Maraviroc for the first 24 weeks in 22 patients. This trial is still enrolling.

Before ART initiation, total HIV DNA in PBMC was significantly lower in Fiebig I versus Fiebig III patients (p = 0.007) and the loss of CD4^+^CCR5^+ ^T cells in sigmoid colonincreased from Fiebig I to III stages (p = 0.0014). After treatment initiation, a rapid and equivalent plasma HIV RNA decline was observed with both regimens.

The percentage of CD4^+^CCR5^+ ^T cells in sigmoid colon significantly increased after antiretroviral treatment in Fiebig III and IV subjects.

Total and integrated HIV DNA in PBMCs declined significantly after Mega-ART and ART. Total HIV DNA by week 24 was undetectable in 40% of patients. Integrated HIV DNA by week 24 was undetectable in 80% of patients.

Total and integrated HIV DNA in sigmoid colon declined significantly after Mega-ART. Total HIV DNA by week 24 was undetectable in 35% of patients. Integrated HIV DNA by week 24 was undetectable in 78% of patients.

The frequency of PBMCs harboring HIV DNA during acute HIV predicted HIV reservoir size after 24 weeks of treatment (p < 0.0001).

Consequently, these studies tend to show that a smaller reservoir size is obtained when ART is initiated very early at acute infection, but no clear benefit is found by adding more than 3 drugs in the combination.

## Purge of the HIV reservoirs

*Zack et al. *[33] proposed a new way to deliver anti-latency agents, like bryostatin, to cells. Following their previous success at incorporating this activator together with Nelfinavir into nanoparticles [34], they proposed to use vaults, which are cellular organelles, as transporters. Consequently, they engineered vaults to include Bryostatin and found a positive effect *in vitro *on reactivation of latent HIV.

*Margolis et al. *[35] presented the first set of results of a trial testing Vorinostat, an HDAC inhibitor. Four patients are currently enrolled in this study which needed a long process of several years for administrative approval. It involves a single 400 mg dose of Vorinostat and looks at HIV expression in resting cells. Initially, the 4 patients were selected as their resting CD4^+ ^cells showed increased viral RNA expression *in vitro *following the presence of Vorinostat. From oncologic studies, it is known that the peak of Vorinostat in plasma occurs between 4 and 8 hours after a single administration. A mean increase *in vivo *of 4.4 fold of resting CD4^+ ^T cell associated full-length RNA was observed after the administration of 400 mg of Vorinostat and sampling patients at around 6 hours post administration.

Provocative data were presented by *Hernandez et al. *[36] suggesting that, beyond its antiretroviral effect, Maraviroc could activate NF-kB in patients regardless of the tropism of their infecting virus. The protocol "TROPISMCV" (NCT01060618) included naïve HIV-infected patients with CCR5-tropic and non-CCR5 tropic viruses, who received a 10 day monotherapy period of Maraviroc. Activity of NF-kB was detected in 4/6 patients with R5 tropic viruses and in 2/3 patients with D/M tropic viruses. This effect may persist in resting cells after withdrawal of the drug in some patients.

*Chen et al. *[37,38] showed that the daphnane diterpene Gnidimacrin activated HIV-1 replication and killed persistently-infected cells at picomolar concentrations *in vitro *in ACH-1 and U1 cells. In these models, Gnidimacrin was at least 2,000-fold more potent than Prostratin.

## ART potency

Whether or not ongoing HIV replication or propagation persists during effective ART is not a new topic, but it has recently been fuelled by the *in vitro *demonstration that ART is quite ineffective at preventing direct HIV cell-to-cell transfer [39].

Bringing the problem at the clinical level, *Schacker et al. *[40] analyzed 12 patients starting a combination with Tenofovir/Emtricitabine and Efavirenz, Atazanavir/ritonavir or Darunavir/ritonavir. Virological and pharmacological measurements were done in PBMCs, inguinal lymph node cells, lymphoid cells from colon and terminal ileum, taken at baseline, months 1, 3 and 6. Concentrations of intracellular drug active forms were measured by UPLC/MS/MS with a limit of detection of 2.5 fmol/10^6 ^cells. Although plasma viremia became undetectable within 2 months in all patients, the authors demonstrated that HIV continued to infect new cells in lymphoid tissues and that the drugs rarely reached effective concentrations in these tissues [41].

It will therefore be important to develop new ways of drug delivery in lymphoid tissues, in particular for strategies purging HIV reservoirs, in order to protect uninfected cells in every compartment.

### Immune modulation

*Deeks et al. *[18] argued that the host response during HIV infection is a barrier to a cure. Inflammation may drive HIV persistence through several non-mutually exclusive mechanisms including negative regulator pathways like the inhibitory receptor programmed death 1 (PD-1). PD-1 plays an active and reversible role in T cell exhaustion and memory CD4^+ ^cells expressing PD-1 contain more proviral DNA than those who do not. Immune therapies based on the blockade of PD-1 interaction with its ligands are supposed to increase HIV production and enhance anti-HIV specific immune responses. The first clinical trial using anti PD-1 antibodies (ACTG 5301) is under approval. It is a single-arm pilot study to evaluate the safety, pharmacokinetic profile, and effects of a single dose of anti-PD-1 antibody in 40 chronically HIV-infected patients receiving effective ART for more than 36 months.

As mentioned previously, *Vandergeeten et al. *[23] presented the potential use of IL-15 to reactivate HIV production from latently-infected cells. IL-15 is 4 fold more potent than IL-7 *in vitro *for reactivating the reservoir while inducing much less cell proliferation. Consequently, IL-15 is a possible candidate to achieve HIV eradication, although IL-7 increases the number of CD4^+ ^T cells harboring HIV integrated DNA.

### Gene therapy

Trying to mimic the "Berlin patient", *June et al. *[42] updated their results with zing finger nuclease (ZFN) to modify CCR5 expression. Six patients with CD4 > 400 cells/mm^3 ^and undetectable viremia received a single infusion of 1 × 10^10 ^modified cells. Four weeks after the infusion, a structured treatment interruption (STI) was planned for a maximum of 12 weeks. Although plasma viremia rebounded in all cases within 4 weeks following the STI, it began to decrease in 5 cases before ART was resumed. In particular, "patient 205" who was heterozygote for the CCR5 delta 32 mutation before ZFN therapy, reached undetectable viral load by day 112. Consequently, ZFN modified cells distributed and trafficked normally compared to endogenous CD4^+ ^cells and showed antiviral activity during STI.

Elimination of CCR5 expression is not expected to directly impact the size of the latent reservoir. If the stability of the latent reservoir is extended by reservoir replenishment, limiting target cell availability would, in turn, reduce potential sources of the virus that drives the replenishment and, in this scenario, an accelerated decay of the latent reservoir would be expected. However if the intrinsic stability of the latent reservoir is indeed measured in decades and there is no replenishment, then introduction of CCR5-negative cells would not alter the decay of the latent reservoir. This should still produce a functional cure since any virus released from reactivated latent cells would be unable to establish new infections. Either scenario would be a significant step forward since patients transduced with CCR5-negative stem cell would be predicted to control the virus in the absence of ART.

*Jerome et al. *[43] have been developing a strategy that employs homing endonucleases with specificity towards conserved HIV sequences. Homing endonucleases able to target sequences within HIV-1 env, pol, tat and vif have been developed. While the emergence of viral mutants with altered cleavage sites are likely to rapidly emerge, investigators are exploring the simultaneous use of several homing endonucleases to avoid the generation of recognition site mutants. These approaches are at a very early stage and numerous obstacles stand in the way of their clinical application. The endonucleases must exhibit exquisite target-site specificity then there is the issue of delivery. Presumably, latently infected cells are only distinguishable from uninfected cells by the presence of an integrated set of viral genes. In the absence of any viral gene expression, it will be difficult to deliver endonucleases (or any interfering gene product for that matter) specifically to infected cells. This would necessitate transducing all permissive cell targets in the host. Nevertheless, the existence of these major obstacles should not stand in the way of exploring any possible strategies with which to eliminate latent or chronically infected cells. Several presentations focused on molecular strategies to limit target cell availability.

## Conclusion

As anticipated, the 5^th ^International Workshop on HIV Persistence led to the presentation and discussion of exciting new data from research groups working towards an HIV cure. Over the years, the control of HIV reservoirs is progressively moving from bench to bedside. The next edition of the workshop will be held in Miami, Fl, 3-6 December, 2013.

## Competing interests

The authors declare that they have no competing interests. The findings and conclusions in this report are those of the authors and do not necessarily represent the views of their institutions.

## Authors' contributions

All the authors contributed equally to the manuscre therapeutic session. All authors read and approved the final manuscript.
